# Microbial drivers of Black Soldier Fly biowaste valorization: from microbiome functions to scalable insect–microbe systems

**DOI:** 10.3934/microbiol.2026005

**Published:** 2026-03-25

**Authors:** Hugo Luttenschlager, Grégoire Noel, Taofic Alabi, Joachim Carpentier, Frédéric Francis, Rudy Caparros Megido

**Affiliations:** Functional and Evolutionary Entomology, URTERRA, Gembloux Agro-Bio Tech, University of Liège, Gembloux, Belgium

**Keywords:** *Hermetia illucens*, microbial consortia, aerobic digestion, composting, anaerobic digestion, frass

## Abstract

Biowaste and agro-industrial co-products continue to increase with population growth and rising living standards, calling for scalable valorization strategies that go beyond simple mineralization. The black soldier fly (BSF) has emerged as a practical bioconversion platform capable of channeling biodegradable organic waste into high-value proteins, lipids, and chitin. In parallel, microbial interventions are increasingly recognized as key levers for substrate conditioning, process stabilization, and performance optimization in BSF-based systems.

In this review, we adopted a function-first perspective to examine how microbial processes shape and connect three major biological valorization routes: aerobic composting, anaerobic digestion (AD), and BSF bioconversion. Rather than focus on taxonomic inventories, we synthesized evidence on microbial functions that matter in practice, including extracellular hydrolysis of complex polymers, regulation of short-chain fatty acids, detoxification and pathogen suppression, and process stabilization. We further reviewed microbe-assisted strategies, such as lactic pre-fermentation, directed acidogenesis, and probiotic or defined consortia and their effects on waste reduction, conversion efficiency, product quality, and sanitary safety.

Finally, we translated these microbial mechanisms into scalable design principles for configuring and operating integrated insect–microbe systems, highlighting how microbial functions underpin reproducible, enterprise-ready performance across composting, AD, and BSF-integrated workflows.

## 1. Introduction

In 2011, roughly one-third of food produced for human consumption was estimated to be lost or wasted [1]. More recent estimates show little or no improvement: ~19% of food available to consumers is wasted at the retail, food-service, and household levels, and ~13.3% is lost between harvest and retail [2],[3]. These food losses represent an economic loss and a loss of agricultural land. Furthermore, it inevitably leads to a demand for the management and recovery of these losses and waste [4]. These measures are essential because poor management of organic waste, particularly food waste, causes numerous well-established health and environmental problems. These include the proliferation of pathogens and pests, the emission of greenhouse gases and odors, soil pollution, and eutrophication of aquatic environments [5]. Two principal biological routes are routinely used to valorize these biowaste streams: composting and anaerobic digestion (AD). Both processes contribute to returning organic matter to soils after aerobic or anaerobic microbial transformation. In this review, AD refers to the biogas-producing process, enabling energy recovery as methane-rich biogas [6].

Composting sanitizes and stabilizes organic matter into compost, a stable organic amendment resulting from controlled aerobic biodegradation. The compost is then applied to soils to restore organic matter accessible to soil fauna and flora. Composting also limits pathogen proliferation and reduces greenhouse gas emissions compared with landfilling [7],[8].

However, specific parameters must be respected, such as the initial C/N ratio and adequate aeration, to achieve these results [9]–[11]. Compost is particularly used as a plant fertilizer. In addition to its physicochemical role as a fertilizer, compost promotes the development of plant-beneficial bacteria at the expense of those that are harmful [12],[13]. Compared to composting, the primary goal of AD is energy production, essentially through methane (CH_4_) production. Like composting, AD can reduce odors and pathogen proliferation and mitigate greenhouse gas emissions. These results should be viewed with caution, as they depend on numerous factors related to the facilities, the materials digested, and the digestion protocols [14],[15]. The resulting digestates, after appropriate storage or treatment, such as composting, can be used as fertilizer [14]. In this way, we can recover a maximum number of simple compounds, redistributing nitrogen, phosphorus, and potassium (N, P, K) to plants and using carbon and hydrogen (C, H) as fuels. Although composting and AD carried out under the right conditions offer better recovery rates and are more sustainable than incinerators or landfills, these waste management techniques have their limitations; for example, composting emits pollutants in the form of greenhouse gases and hydrogen sulfide. Furthermore, the production of synthetic fertilizers remains much faster than the composting process [16]. Regarding AD, the accumulation of heavy metals and the production of digestate classified as immature compost also pose problems in terms of completing the recovery of organic matter [17].

New valorization methods aim to convert biodegradable organic waste into valuable, complex organic molecules such as lipids and proteins. The black soldier fly (BSF; *Hermetia illucens* (L. 1758)), at its larval stage, is a voracious and polyphagous insect capable of processing a wide range of biodegradable substrates, including food waste, agro-industrial by-products, garden residues, and animal manures. The BSF is mostly used in animal feed, in monogastrics, and aquaculture farming because of its protein content being generally around 40% (dry matter) and its good amino acid profile [18]–[22]. However, it is also of interest for lipid and chitin production, which can be used in animal feed and in industry [23],[24]. Moreover, the material that the larvae digest but do not assimilate is valuable. The residues of organic matter and insect excrement, called frass, can be used as pre-compost or fertilizer [25]. Numerous studies highlight that the gut microbiome of the BSF plays a crucial role in its ability to degrade and digest food through enzymatic activity [26]–[28]. These discoveries led to researchers investigating the impact of microorganisms, such as gut-associated, substrate-associated, or inoculant microorganisms, on the growth and composition of edible insects, including *Tenebrio molitor* L. 1758, and, notably, *H*. *illucens* [23].

Classical biowaste treatments such as aerobic composting and AD are fundamentally mineralization-driven: Microorganisms depolymerize complex organic matter and convert carbon to simple inorganic end-products (CO_2_ or CH_4_), while nitrogen appears as ammonium and nitrate, yielding a stabilized residue (mature compost or digestate) [29],[30]. By contrast, BSF bioconversion introduces a dual pathway, in which a fraction of carbon and nitrogen can be recaptured into harvestable larval biomass, while substantial mineralization occurs through larval respiration and associated microbial activity, particularly under waste reduction–oriented or low-quality substrate conditions.

In this review, we move beyond descriptive taxonomic surveys to adopt a function-first perspective on microbial roles across composting, anaerobic digestion, and BSF bioconversion, with particular emphasis on process interfaces and scalability. The term “integrated BSF–microbial systems” does not imply that microorganisms are optional components of BSF rearing. Rather, it explicitly recognizes that microbial activity is inherently coupled to BSF larvae feeding on moist organic substrates. We use “integrated” to distinguish (i) implicit, unmanaged microbial processes that occur in all BSF systems and (ii) deliberately designed or steered microbial functions (e.g., substrate preconditioning, fermentation control, pathogen suppression) that are intentionally leveraged to improve process performance and reproducibility. Focusing on food-related biowaste (food waste and agro-industrial by-products), we first establish the microbial architecture of the two classical valorization routes, aerobic composting (Section 1) and anaerobic digestion (Section 3), before examining how BSF larvae integration reshapes microbial succession, carbon partitioning, and process kinetics (Sections 2 and 4). We then synthesize the functional roles of gut-associated and substrate-associated microbiota in BSF-mediated bioconversion (Section 5), emphasizing four key mechanisms: extracellular hydrolysis of complex polymers, modulation of short-chain fatty acids, detoxification and pathogen suppression, and effects on substrate physical structure. Finally, we consider practical integration pathways, microbial substrate conditioning upstream of BSF rearing, and downstream frass valorization via composting or anaerobic digestion, alongside scalable design principles for configuring and operating the insect–microbe holobiont toward reproducible, enterprise-ready performance (Section 6).

## 2. Composting

Composting is a well-known biological strategy for stabilizing biodegradable organic waste, including food waste and agricultural by-products. It is composed of a four-stage aerobic process driven by microbial succession: the first mesophilic phase, the thermophilic phase, the second mesophilic phase or cooling phase, and the maturation or curing phase [31],[32].

During the first mesophilic phase, temperature reaches ~45 °C, and heterotrophic microorganisms, including bacteria (notably actinomycetes) and fungi, rapidly colonize the substrate. These microorganisms start to hydrolyze the most accessible fractions, including soluble sugars, starch, proteins, and lipids. These microorganisms secrete extracellular hydrolases and oxidative enzymes such as cellulases and xylanases (targeting cellulose and hemicellulose), proteases (releasing peptides and amino acids and initiating nitrogen mineralization), lipases (driving triacylglycerol breakdown into free fatty acids and glycerol), and amylases (converting starch into maltose, dextrins, and glucose). Lignin-modifying enzymes (laccases, peroxidases) can be detected at this early stage, initiating the opening of lignocellulosic structures [33],[34].

The heat generated during the transitions into the thermophilic phase (50–70 °C) dries the substrate and suppresses most opportunistic pathogens. High temperatures indicate thermophilic and thermotolerant decomposers that continue polymer breakdown using heat-stable cellulases, xylanases, lignocellulolytic oxidases, and thermostable proteases and lipases [31],[35],[36]. Although lignin depolymerization is traditionally attributed to fungi, the thermophilic core community is primarily composed of bacteria, reflecting higher thermal tolerance and rapid turnover [37]. Dominant taxa frequently include spore-forming Bacillaceae (*Bacillus, Paenibacillus, Geobacillus*), Proteobacteria, such as *Pseudomonas*, *Acinetobacter*, *Enterobacter*, *Aeromonas*, and *Xanthomonas*, and thermophilic or thermotolerant actinomycetes (*Streptomyces, Rhodococcus, Micromonospora, Thermomonospora, Nocardia*) [35],[37].

The second mesophilic phase, or cooling phase, is characterized by a drop in temperature below 45 °C. This temperature enables mesophilic microorganisms to recolonize the compost [38],[39]. During this phase, nitrogen mineralization continues, but the dominant fate of inorganic nitrogen shifts toward retention as nitrite and nitrate via nitrification rather than loss as volatile ammonia. Humification begins during the cooling phase: Partially oxidized lignocellulosic fragments and other organic intermediates are incorporated into humic-like structures [29].

Finally, the maturation or curing phase is defined by the compost returning to ambient temperature and the biochemical stabilization of the remaining organic matter into humic substances. Humification is the progressive transformation of residual organic compounds such as structural polysaccharides, soluble carbohydrates, proteins, and lipids into humic acids, fulvic acids, and humin [40]. These humic substances are crucial in mechanisms that retain nitrogen within the final compost matrix, thereby limiting nitrogen losses and improving agronomic value [41]. This extended curing phase is primarily driven by saprotrophic fungi and actinomycetes, which start to dominate once thermophilic bacteria decline.

The intrinsic parameters of compost strongly influence the composting process. Temperature, moisture, pH, aeration, particle size, C/N ratio, and nutrient availability directly shape the development and activity of the composting microbiota [42],[43]. Beyond these intrinsic factors, introducing BSF larvae acts as an additional biological lever that can modify process trajectories.

## 3. Black soldier fly integration with aerobic digestion

**Figure 1.**
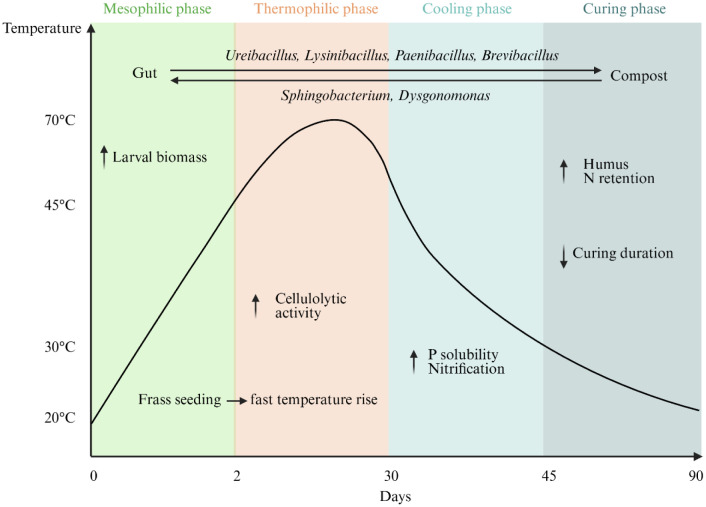
Conceptual schematic. Composting temperature curve (mesophilic, thermophilic, cooling, maturation) with two BSF windows: (i) An optional mesophilic pre-treatment at the very start (≤ ~35–40 °C), sometimes used to jump-start degradation (via frass or larvae); (ii) an operational window at the thermophilic → cooling transition (≤ ~45 °C), where introducing larvae has been associated with faster nitrification, shorter curing, enhanced humification, and in some cases, higher P solubility. No larvae are applied during the thermophilic core (50–70 °C), which is too hot for BSF. Bidirectional microbiome exchange is indicated (*Ureibacillus* from gut to compost; *Sphingobacterium* from compost to gut). Frass roles: Low-temperature seeding, co-composting (more humus, greater N retention), and post-composting as a finishing step for stability and hygiene. Created in BioRender. Luttenschlager, H. (2026) https://BioRender.com/wgxqolf

BSF–composting integration occurs either simultaneously, larvae introduced into an active pile, or sequentially via frass post-composting; both routes remain aerobic but shift process trajectories and endpoints. Compared with conventional composting, BSF assisted composting increases the bioavailable carbon pool via larval biomass accumulation and enhances humification [44]. It also enriches cellulolytic activities, reflected by higher relative abundances of bacteria associated with polysaccharide breakdown [45]. Several studies show that adding BSF larvae to the compost accelerates nitrification and shortens the curing phase [46]. In parallel, the compost microbiome often gains Bacillota (Firmicutes) taxa, including *Ureibacillus*, *Lysinibacillus*, *Paenibacillus*, and *Brevibacillus*, which are abundant in the larval gut; conversely, substrate-derived bacteria such as Moheibacter (Bacteroidota) and Actinomadura (Actinomycetota) can colonize the larval microbiome [46]. Altogether, these patterns indicate a two-way bacterial exchange between the substrate and the gut, and a strong substrate effect on gut community structure and function, with *Sphingobacterium* and *Dysgonomonas* frequently dominant in BSF intestines [47]. Beyond humification and nitrification, a one-week BSF composting step applied after 30 days of thermophilic composting can also increase phosphorus solubility, a response associated with the dominance of *Nocardiopsis* and *Pseudomonas* during the BSF phase [48] (Figure 1). Notably, evidence shows that gut communities are shaped more by the substrate physical structure, especially particle size, than by the substrate's bulk microbiota; particle-size standardization and reporting should therefore accompany any microbial inoculation or pretreatment claims [49].

Although BSF associated microorganisms can, by themselves, improve composting performance, targeted inoculations can further enhance composting or larval development. On banana peels, microbial pretreatments, particularly *Rhizopus oligosporus* for ~14 days, alone or combined with non-protein nitrogen, significantly increase larval performance and protein yield per kilogram of incoming material [50]. Moreover, a combined thermal/physical (milling) pretreatment plus microbial consortium (Actinomycetales, Caryophanales, Cellvibrionales, Clostridiales, Desulfobacterales, Eubacteriales, Micrococcales, Pseudomonadales, Sphingobacteriales) recondition the substrate and lead to significant reductions in larval lipid and saturated fatty-acid contents, indicating compositional steering of the harvested biomass [51].

Frass is typically an immature compost and not always suitable for direct use as fertilizer [52]. Post-composting can improve its stability and degree of humification [53]. Nonetheless, adding frass to the composting process can boost humification and enhance nitrogen retention in the final product [54]. Mechanistically, frass, particularly its Sphingobacteriaceae members, can initiate low-temperature composting and raise temperatures, facilitating subsequent colonization by other bacterial guilds; frass-borne taxa such as Cellulomonadaceae and Halomonas have been associated with higher humus formation in co-composts [55] (Figure 1).

## 4. Anaerobic digestions

Hydrolysis is the first stage of anaerobic digestion and is carried out by hydrolytic bacteria, mostly within Bacteroidetes, Firmicutes, and Proteobacteria. In this stage, particulate organic matter is solubilized by a broad suite of extracellular hydrolases that depolymerize proteins, lipids, starch, and structural polysaccharides into bioavailable intermediates [56]. These enzymes solubilize particulate organic matter into smaller, bioavailable intermediates: amino acids and peptides from proteins, free long-chain fatty acids and glycerol from triglycerides, and oligosaccharides and simple sugars from cellulose, hemicellulose, and starch. Hydrolysis is generally considered the rate-limiting step for anaerobic digestion of complex organic wastes, because downstream microbial guilds cannot access particulate material directly if it is not solubilized [57].

The soluble products released during hydrolysis then serve as substrates for the next stage, acidogenesis, where they are rapidly fermented into volatile fatty acids, hydrogen, and carbon dioxide. The dominant acidogenic guilds in food-waste anaerobic digesters are typically Firmicutes of the class Clostridia, which perform mixed-acid fermentation by converting pyruvate to acetyl-CoA and then to acetate via phosphotransacetylase/acetate kinase, concomitantly releasing H_2_ and CO_2_ [58]–[60]. Bacteroidetes also contribute substantially to acetate and propionate formation, especially in carbohydrate-rich substrates, by coupling polysaccharide breakdown to short-chain fatty acid production. In the very early stages, facultative anaerobes within the Proteobacteria can transiently dominate, rapidly producing acetate, lactate, and ethanol and lower pH, which favors the subsequent establishment of strict anaerobic Clostridia [59].

In contrast to acidogenesis (which forms a mixed VFA pool), acetogenesis syntrophically oxidizes propionate/butyrate/alcohols into acetate, CO_2_, and H_2_ [61]. Because these conversions are endergonic at elevated H_2_ partial pressure, accumulation of H_2_ inhibits the activity of syntrophic acetogenic bacteria [62]. For acetogenesis to proceed, tight syntrophy with H_2_-scavenging methanogenic archaea is essential; by consuming H_2_, methanogens keep hydrogen partial pressure low and render the reactions thermodynamically favorable [63]. Key syntrophic acetogens include *Syntrophomonas* (butyrate/long-chain fatty-acid oxidizer) and *Syntrophobacter* and *Pelotomaculum* (propionate oxidizers) [61],[64].

Methanogenesis is the terminal stage of anaerobic digestion, carried out exclusively by archaeal methanogens and culminating in CH_4_ production. The dominant methanogenic pathway depends on digester conditions. The hydrogenotrophic route operates when the H_2_ partial pressure is kept low, converting H_2_ and CO_2_ into CH_4_ and H_2_O (Oliveira et al., 2025). The aceticlastic route, restricted to the genera *Methanosarcina* and *Methanothrix* (formerly Methanosaeta), converts acetate directly into CH_4_ and CO_2_ [65]. Finally, the methylotrophic route, less common in food-waste management, reduces methanol, methylamines, and methyl sulfides to CH_4_ [66].

## 5. Black soldier fly integration with anaerobic digestion

Compared at comparable scales, anaerobic digestion recovers approximately 53% of food-waste carbon as methane (667 mL CH_4_ g⁻¹ volatile solids), thereby prioritizing energy recovery. In contrast, BSF converts approximately 29% of carbon, 34% of nitrogen, and 33% of phosphorus into harvestable larval biomass (309 g kg⁻¹ dry food waste at pilot scale), reflecting a material-oriented recovery of nutrients [67]. These differences do not merely indicate divergent efficiencies, but fundamentally distinct carbon partitioning pathways; energy recovery via methanogenesis versus nutrient recapture into biomass, thereby underpinning complementary resource-recovery profiles.

One way to operationalize this complementarity is to use digestate from AD as a substrate for BSF rearing. Larval performance and nutritional composition depend strongly on substrate quality. In some cases, BSF larvae reared on food-waste digestate show faster development and higher protein content than larvae fed the same food waste without prior AD [68]. However, when the AD residue originates from low-quality or recalcitrant feedstocks, such as yard trimmings, agricultural residues, or certain manures, larval growth can be inferior to that obtained on standard control diets [69]–[71].

To our knowledge, no researcher has combined deliberate microbial inoculation inside the methanogenic reactor with the dual aim of increasing methane yields and simultaneously improving the downstream digestate as a BSF feed. By contrast, several researchers have explored substrate fermentation (with or without added inocula) outside AD to enhance BSF diet quality [72],[73]. Lactic fermentations generally improve larval growth, and while *Lactobacillus spp* are often associated with positive outcomes, explicit inoculation with lactic acid bacteria is not always necessary; likely because these bacteria are ubiquitous in food waste matrices [72],[73]. Co-fermentation studies on vegetable waste further show that shifts in fermentation profiles are tightly coupled to changes in bacterial community composition, reinforcing the role of substrate conditioning rather than inoculation per se in shaping downstream bioconversion performance [74].

**Figure 2.**
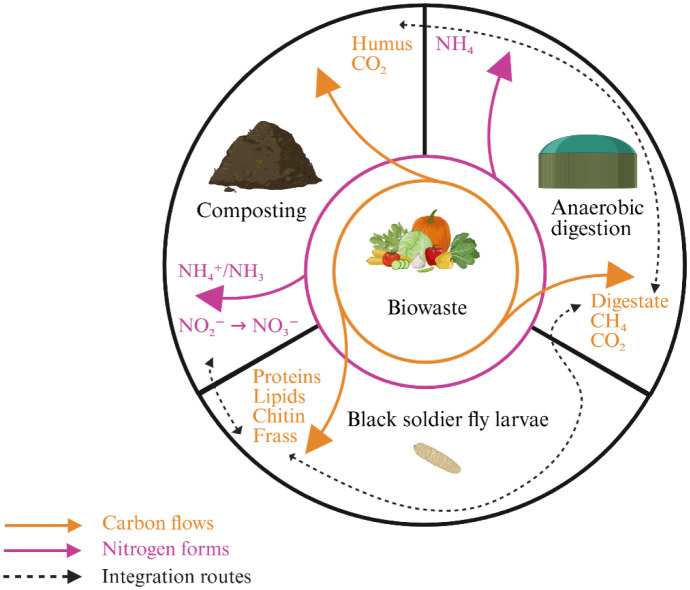
Conceptual carbon–nitrogen cycle across composting, anaerobic digestion, and black soldier fly larvae bioconversion. Orange: carbon flows (compost → CO_2_ and humus; AD → CH_4_/CO_2_ and digestate; BSF → larval biomass: proteins, lipids, chitin, and frass). Purple: nitrogen forms (compost: NH_4_⁺/NH_3_ → NO_2_⁻ → NO_3_⁻ during cooling/maturation; AD: digestate NH_4_⁺ nitrifies after soil application or post-composting). Dashed arrows: integration routes (frass → compost; frass → AD; digestate → compost; digestate → BSF, if suitable). Operational note: BSF window ≤ 30 °C at the thermophilic → cooling transition; no BSF in the thermophilic core (50–70 °C). *Schematic, not to scale*. Created in BioRender. Luttenschlager, H. (2026) https://BioRender.com/jlz5y3z.

A second BSF/AD interface is the frass-to-AD pathway, in which larvae first predigest the feedstock, and the resulting frass is then anaerobically digested for energy recovery. Although this valorization route is less common, adopted by ~12.5% of European insect farms versus ~75% that apply frass as fertilizer, available studies indicate that frass can deliver methane yields comparable to livestock manures [75],[76]. As far as we are aware, no defined bacterial or archaeal strains are used as primary inocula to improve AD of frass; instead, researchers rely on complex inocula (anaerobic sludge/digestate) carrying the whole microbial consortium required for hydrolysis, acidogenesis, acetogenesis, and methanogenesis [77]. Finally, whether it is a digestate of frass or frass resulting from BSF consumption of a digestate, it is generally necessary to apply a composting step [78] to stabilize the organic matter (Figure 2).

## 6. Microbiota-mediated biochemical pathways in BSF food waste bioconversion

Advances in high-throughput sequencing have greatly expanded our understanding of insect gut microbiomes, including BSF [79]–[81]. BSF feed on microbe-rich substrates, and their gut microbiota performs essential functions in larval development and food waste degradation while protecting against pathogens and xenobiotics [82]. These microbes, including bacteria and fungi, contribute to macronutrient breakdown, such as starch hydrolysis in the anterior midgut, thereby reducing the nutrients available for microbial metabolism [83]. While BSF rely on gut microbes for digestion, studies show no globally conserved core microbiome [81],[84]; instead, a few bacterial genera, such as *Dysgonomonas* spp., *Enterococcus* spp., *Klebsiella* spp., and *Providencia* spp., occur more frequently in degrading carbohydrates, proteins, lipids, and mineral-rich compounds (Table 1). The microbiome composition of BSF varies with diet [85]–[87]; thus, with the given bio-waste, environmental factors (e.g., geography), and rearing conditions, and microbe functional importance does not always correlate with the dominance of specific microbial taxa. This dynamic interaction highlights the microbiome's influence on BSF adaptability and efficiency in its food waste conversion. However, some core microbiomes are favored for degrading specific dominant biochemical compounds in food waste products, such as domestic or canteen waste [81],[87]–[89]. While bacterial communities dominate current research, fungal taxa, mainly Ascomycota, emphasize the bioconversion of more lignified biowaste [27],[90]. Additionally, biological pretreatments, such as white-rot fungi or *Trichoderma reesei*, enhance substrate digestibility and stimulate microbial enzyme activity, improving BSF bioconversion [50],[91]. Similarly, bacterial pretreatments with *Bacillus subtilis* can accelerate lignocellulosic co-waste degradation [92].

Studies, mainly on bacterial communities (Table 1), reveal that microbial abundance increases toward the hindgut while diversity declines [93], which is likely due to selective pressures in the midgut and favorable conditions in the hindgut [94]. However, functional understanding of microbiome-mediated processes remains limited (Table 1), as most researchers rely on predictive approaches such as PICRUSt (*Phylogenetic Investigation of Communities by Reconstruction of Unobserved States*) [95],[96] rather than direct experimental evidence demonstrating causal microbial contributions to the degradation of specific biowaste components [97]. High-fiber substrates, including domestic biodegradable waste, coffee silverskin, and agricultural residues, selectively enrich cellulolytic and hemicellulolytic bacteria such as *Dysgonomonas*, *Actinomyces*, and *Cellulomonas*. These microbes express carbohydrate-active enzymes (CAZymes), including cellulases, hemicellulases, and β-glucosidases, which hydrolyze cellulose and hemicellulose into fermentable sugars. In lignocellulose-rich diets, frass microbiota further contribute by producing ligninases, facilitating the breakdown of recalcitrant lignin polymers [93],[97]. This enzymatic synergy accelerates fiber depolymerization, enhancing carbon flux toward larval metabolism. Protein-rich substrates, such as chicken manure and mixed food waste, mostly promote taxa such as *Enterococcus* and *Klebsiella*, which harbor genes for amino acid catabolism and nitrogen cycling. These microbes participate in deamination and ammonification processes, releasing nitrogenous compounds that support larval growth and microbial biosynthesis [67],[98]. The presence of urease and protease activities within these communities underscores their role in converting complex proteins into peptides and free amino acids, optimizing nitrogen assimilation. Lipid-rich food wastes, including oilseed meals and domestic residues, induce microbial succession toward taxa such as *Corynebacterium* and *Marinobacter*. These bacteria enhance fatty acid β-oxidation and lipase-mediated hydrolysis, preferentially degrading medium-chain fatty acids over long-chain fatty acids and sterols [99]. This process not only accelerates lipid turnover but also supports the synthesis of microbial and larval lipids, thereby improving the energy density of BSF biomass [100]. Diets supplemented with biochar or superphosphate recyclates enrich phosphate-solubilizing microbes, such as *Pseudomonas*, which mobilize inorganic phosphorus through organic acid secretion and phosphatase activity [101]. This enhances mineral bioavailability, supporting larval development and microbial enzymatic functions. Similarly, co-treatment of chicken manure with food waste reshapes microbial communities to optimize carbohydrate, lipid, and amino acid metabolism, demonstrating the potential of microbiota engineering for improved bioconversion [98].

Fungal communities, although less studied than bacterial counterparts, play an essential role in BSF-mediated food waste conversion. Yeasts such as *Cyberlindnera* and *Pichia* have been identified in the larval gut when fed mixed agricultural wastes, including brewers' spent grain, kitchen waste, poultry manure, and rabbit manure, suggesting a potential contribution to fermentation processes and metabolite production that support bacterial growth and enzymatic activity [90]. Beyond gut-associated fungi, substrate pretreatment using lignocellulose-degrading fungi has been shown to influence microbial ecology significantly; for example, fungal conditioning of palm oil industry side streams (empty fruit bunches and palm kernel meal) shift the larval gut microbiome toward cellulolytic bacterial taxa, thereby enhancing fiber degradation efficiency [102],[103]. These findings indicate that fungi act as direct symbionts and as ecosystem engineers by reducing substrate complexity and improving accessibility for bacterial cellulases and hemicellulases, such as those in the genera *Dygonomonas* and *Actinomyces* [95],[97].

**Table 1.** Overview of researchers investigating associations between biowaste type, dominant BSF gut microbial taxa, major biochemical fractions of the substrate, and molecular methods.

Used biowaste	Identified core microbiota driven by biowaste	Main biochemical compound of the biowaste	Used molecular methods	Reference
Restaurant food waste and household food waste	*Enterococcus* spp., *Dysgonomonas* spp., *Actinomyces* spp., *Klebsiella* spp.	Carbohydrates, proteins, nitrogenous compounds and lipids	Metabarcoding V3-V4 region of 16S rRNA; functional prediction from 16S metabarcoding data	[95]
Coffee silverskin residue	*Actinomyces* spp., *Dysgonomonas* spp.	Lignocellulose	Metabarcoding V3-V4 region of 16S rRNA	[93]
Edible mushroom residues, soybean residue, pig concentrated feed, pig concentrated feed with enrichment of cellulose	*Morganella* spp., *Enterococcus* spp., *Paenibacillus* spp., *Klebsiella* spp., genera of Enterobacteriaceae	Fibers	Metabarcoding V3-V4 region of 16S rRNA	[86]
Chicken manure, kitchen waste	*Enterococcus* spp., *Bacillus* spp., *Lactobacillus* spp.	Protein, lipid	Metabarcoding V3-V4 region of 16S rRNA	[104]
Chicken feed, high-fiber diet, high-fiber enriched with biochar, high-fiber enriched with single superphosphate recyclate	*Morganella* spp.	Fibers, lipids, proteins, minerals	Metabarcoding V3-V4 region of 16S rRNA; Quantification of specific taxa	[101]
Food waste, supermarket food leftovers, fruit waste, fruit waste with soybean residue, peanut coat with soybean residue	*Providencia* spp., *Entorococcus* spp., *Klebsiella* spp.	Fibers, lipids, proteins, minerals	Metabarcoding V3-V4 region of 16S rRNA	[87]
Domestic biodegradabke waste	*Lactobacillus* spp., *Dysgonomonas* spp., *Prevotella* spp., *Cellulomonas* spp., *Corynebacterium* spp.	Hemicellulose, cellulose and lignin	Metabarcoding V3-V4 region of 16S rRNA; carbohydrate-active enzymes (CAZymes)	[97]
Empty fruit bunches, potato pulp, cottonseed press cake	Bacillaceae spp., *Cellulomonas* spp., *Bacillus* spp.	High-fiber diets	Sanger-based 16S rRNA gene sequencing of pure isolates; functional prediction from 16S metabarcoding data; Screening of cellulose-degrading strains	[103]
Oilseed meal supplementation at 20% for flaxseed meal, tiger nut meal, and Zanthoxylum bungeanum seed meal	Mainly Firmicutes, unclassified Lachnospiraceae, *Entorococcus* spp., *Clostridioides* spp. and *Klebsellia* spp.	Lipid	Metabarcoding V3-V4 region of 16S rRNA	[100]
Domestic biodegradable waste	*Corynebacterium* spp., *Marinobacter* spp., *Brevibacterium* spp.	Lipid	Metabarcoding V3-V4 region of 16S rRNA; functional prediction from 16S metabarcoding data	[99]
Co-treating agri-food waste corresponding to a 7:2 chicken manure-to-food waste ratio	*Enterococcus* spp.	Fibers, lipids, proteins, minerals	Metabarcoding V3-V4 region of 16S rRNA	[98]
Brewers' spent grain, kitchen food waste, poultry manure, and rabbit manure	*Cyberlindnera* spp., *Pichia* spp.; *Dysgonomonas* spp., *Morganella* spp., *Enterococcus* spp., *Pseudomonas* spp., *Actinomyces* spp., *Providencia* spp.	Fibers, lipids, proteins, minerals	Metabarcoding V3-V4 region of 16S rRNA and ITS2	[90]
Chicken feed, chicken manure, and camelina substrate composed of 50% chicken feed and 50% camelina oilseed press cake	Planococcaceae (unassigned genus), Bacillaceae (uncultured), *Bacillus* spp., *Providencia* spp., *Lactobacillus* spp.	Fibers, lipids, proteins, minerals	Metabarcoding V3-V4 region of 16S rRNA	[85]
Two palm oil industry side streams: empty fruit bunches and palm kernel meal	*Trichosporon asahii*, Klebsiella spp., *Enterococcus* spp.	Cellulose, Hemicellulose, Lignin, Protein, Fiber, residual lipid	Metabarcoding V3-V4 region of 16S rRNA and ITS2; functional prediction from 16S metabarcoding data	[102]
Chicken feed, from canteen to food waste and oil waste diets	*Morganella* spp., *Acinetobacter* spp., *Dysgonomonas* spp., *Providencia* spp.	Fibers, lipids, proteins, minerals	Metabarcoding V3-V4 region of 16S rRNA ; functional prediction from 16S metabarcoding data	[88]
Postproduction soy pulp and postconsumer cafeteria waste	*Dysgonomonas* spp., *Porphyromonas* spp., *Parabacteroides* spp.	Fibers, lipids, proteins, minerals	Metabarcoding V3-V4 region of 16S rRNA	[89]

## 7. Integrated BSF–microbe waste valorization: implications of microbial functions for scale-up and policy

This perspective extends the microbial mechanisms discussed above by examining how microbial functions (hydrolysis, acidification, pathogen suppression, and nutrient stabilization) translate into system-level design choices, regulatory constraints, and industrial deployment of BSF–microbe valorization platforms.

The global escalation of organic waste generation, particularly biodegradable organic waste streams relevant to biological valorization, represents a severe environmental challenge and a largely underexploited economic opportunity. Biological waste valorization strategies based on insects and microorganisms are increasingly recognized as scalable approaches that can convert organic residues into high-value bioproducts while reducing greenhouse gas emissions and resource losses. This perspective argues that integrated systems combining BSF larvae with microbial processes should be viewed not merely as waste treatment technologies, but as modular platforms for circular bioeconomy enterprise development. Drawing on scientific evidence and industrial-scale implementations in Europe (Protix), North America (Enterra), and Africa (ProtiCycle), we illustrate how BSF–microbial systems can support value chains in animal feed, biofertilizers, and bioenergy. Key technological, regulatory, and financial enablers are discussed, and strategic research and policy priorities needed to accelerate commercialization are outlined.

### 7.1. Reframing organic waste as a strategic bioeconomic resource

The generation of organic waste is increasing worldwide, with biodegradable organic waste constituting a major and growing fraction of this stream due to food loss and agro-industrial activities [105]. For example, the food waste sector accounts for 931 million tons per year [106]. This fraction is associated with several gigatons of CO_2_-equivalent emissions, with estimates commonly around 3.3 Gt CO_2_-equivalent, when upstream production losses and downstream waste management are considered [107]. Conventional waste management approaches, including landfilling and incineration, are increasingly unsustainable. They contribute substantially to greenhouse gas emissions, nutrient losses, and rising operational costs [108].

Within circular bioeconomy frameworks, organic waste is increasingly reconceptualized as a renewable feedstock that supports productive economic cycles. Biological conversion systems are particularly attractive because they combine waste reduction with resource recovery. Among these systems, insect-based bioconversion using BSF and microbial degradation processes have emerged as scalable and complementary solutions [109].

This perspective advances the view that integrated BSF–microbial systems constitute a foundational technological platform for a new class of circular bioeconomy enterprises, rather than a niche waste management intervention.

### 7.2. Strategic rationale for integrating *H. illucens* and microbial systems

The larvae of *H. illucens* can rapidly assimilate heterogeneous biodegradable organic waste streams, including food waste, market residues, and agro-industrial by-products. Empirical studies consistently report substantial reductions in waste mass over short processing periods. This efficiency reduces reliance on landfills and associated greenhouse gas emissions [105],[108].

Compared with composting or anaerobic digestion, BSF-based systems require smaller land footprints and enable faster biomass turnover. These characteristics make them particularly suitable for urban and peri-urban environments, where space is often limited [109].

Microbial systems complement insect bioconversion by enabling substrate hydrolysis, detoxification, and nutrient solubilization. Through mineralization and pathogen suppression, microbial consortia enhance feedstock stability and expand the range of waste substrates that can be effectively valorized [110]. Importantly, microbial activity does not exclusively benefit BSF larvae. Depending on process conditions, microorganisms may also compete with larvae for readily available nutrients, potentially reducing feed conversion efficiency and larval biomass yields. While such competition may be acceptable or desirable in waste reduction-oriented systems, it becomes a critical design constraint when maximizing larval productivity is a primary objective.

Introducing BSF larvae at the late thermophilic/early cooling transition aligns with faster nitrification, shorter curing, enhanced humification, and sometimes higher P solubility [46],[47]. These outcomes coincide with two-way microbiome exchange (e.g., Bacillota from gut to pile; and *Moheibacter/Actinomadura* from substrate to gut) and are strongly conditioned by substrate physical structure, notably particle size, which should therefore be standardized and reported [47]. Adding frass can also seed low-temperature activity, a helpful feature in temperate settings and a prompt to identify BSF-associated microbes that best support low-T composting [55].

### 7.3. From waste treatment to multi-output value creation

Integrated BSF–microbial systems generate multiple, economically distinct outputs. BSF larvae produce protein- and lipid-rich biomass suitable for animal feed, aquaculture, and pet food markets. Larval frass can be commercialized as a nutrient-rich organic fertilizer [111]. Insect-derived oils and chitin further extend value creation into bioenergy and biomaterial markets [108]. Even before accounting for any value-added products generated by BSF bioconversion, the waste-management service (organized collection and sanitary treatment of pathogen-bearing organic residues) have stand-alone economic value (gate fees, avoided disposal costs) and public-health benefits [112].

To date, there are no well-documented reports of BSF production systems experiencing major sanitary incidents, either through acquisition of pathogenic fungi, viruses, bacteria, or protozoa, or through transmission of such pathogens to humans or livestock, under standard rearing conditions [113]. This apparent robustness is often attributed to a competent innate immune system and to the resident gut/substrate-associated microbiota that colonize BSF rearing environments [113]. Experimentally, BSF processing has reduced counts of selected pathogens in manures, including *Bacillus*, *Salmonella*, *Vibrio*, and *Enterococcus* [114]. On kitchen-waste matrices, BSF activity has likewise been associated with decreases in bacterial genera such as *Lactobacillus*, *Devosia*, and *Pseudomonas* in the residual substrate [115].

Because BSF plus their associated microorganisms can lower, but not necessarily eradicate, pathogens in diverse feedstocks (AD sludges, compost inputs, food/agro-industrial residues subsequently composted), there is a rationale to systematically couple BSF with defined “core” gut/consortial microbiota to optimize sanitization performance across substrates and process windows [90],[116],[117].

From a business perspective, this diversity of outputs improves revenue stability and reduces exposure to market volatility. In practice, post-composting of frass and/or digestate often provides a low-cost final polishing, improving stability, odors, hygiene, and nitrification-driven N retention, before land application.

### 7.4. Industrial evidence supporting commercial viability

Industrial-scale facilities demonstrate that BSF–microbial systems can operate at a commercially meaningful scale. In Europe, Protix operates a flagship insect production facility in Bergen op Zoom (Netherlands). The facility reportedly produces approximately 14,000–15,000 tons of live BSF larvae annually using food processing residues [118].

In North America, Enterra Feed Corporation operates a facility in Alberta (Canada) capable of processing more than 130 tons of pre-consumer food waste per day. The operation produces insect-based feed ingredients and organic fertilizers [119].

Together, these cases confirm that BSF–microbial systems can support viable waste-to-value business models. They also validate earlier conceptual frameworks linking waste treatment services with bio-based product markets [109]. Reported throughputs and product splits are site-specific; substrate portfolios, preprocessing, and regulatory constraints strongly modulate transferability.

### 7.5. Enabling conditions for enterprise development and scale-up

The transition from pilot projects to industrial deployment depends on several enabling conditions. Public financing and policy support are significant during early commercialization. For example, approximately CAD 6 million in federal funding supported Enterra's expansion in Canada [120]. Regulatory clarity is equally critical. Rules governing insect-based feed, standardized bio-safety protocols, and robust traceability systems reduce investor risk. Advances in automation, digital monitoring, and microbial process control are expected to improve operational efficiency and economic performance further.

In addition to biological optimization, several digital and automation tools are being explored in BSF production systems, including near‑infrared spectroscopy to monitor feed, larvae, and frass quality [121], real‑time computer‑vision pipelines for automated larval stage classification [122],[123], and IoT‑based incubators that continuously log and regulate environmental conditions during BSF rearing [124].

### 7.6. Challenges and strategic research priorities

Evidence supports a function-first view: Diet and substrate structures select guilds (cellulolytic/hemicellulolytic, proteolytic/ureolytic, lipolytic/β-oxidation, phosphate-solubilizing) whose enzyme portfolios (CAZymes, proteases/ureases, lipases, phosphatases) drive conversion more than any single “core” taxon [95],[97]–[99],[101]. Priority work should move beyond predictive metagenomics (e.g., PICRUSt) toward causal validation (direct enzyme assays, enrichment/removal of guilds, gnotobiotic or defined-consortium trials) linked to larval outcomes (growth, protein/lipid quality).

Despite their potential, BSF–microbial systems face several challenges, including regulatory fragmentation, risks of heavy metal accumulation, and pathogen management [81],[125]. However, a crucial issue that arises in composting or AD is the lack of industrial-scale implementation and standardization of the methods and equipment used in these experiments. Comparing studies using BSF to improve the composting process is difficult due to the lack of standardization of the feedstock used, varying C/N ratios, varying treatment times, and the lack of certain information such as particle size or substrate aeration [44]–[46]. Although some studies on frass composting can be conducted on a large scale, there is difficulty in comparing these studies due to the feed or coproducts used, or variables such as composting temperature [53]–[55]. A significant challenge in developing the integrated use of BSF and AD does not lie in the maturity of the individual technologies. Though, composting and AD are well-established at the industrial scale, they are limited in the validation of their interconnection at full scale. While scalability of BSF-based processes from laboratory to pilot scale has been demonstrated [67], the literature lacks industrial-scale data on coupled BSF–AD systems. Rather than enforcing rigid experimental standardization, future progress would benefit from standardized reporting frameworks and reference benchmarks that preserve experimental innovation while enabling meaningful comparison across studies [68],[70],[126]. Similar standardization challenges arise when BSF frass is used as a substrate for AD, particularly with respect to scale, feed characteristics, and pre-treatment steps such as drying or conditioning prior to digestion [75],[76].

Furthermore, in future studies, researchers should focus on microbial–insect interactions, life-cycle performance, and long-term economic viability. From an enterprise perspective, priority areas include cost reduction, modular system design, digital integration, and alignment with waste and agri-food infrastructures.

### 7.7. Outlook: Toward a new circular bioeconomic industry

This perspective argues that integrated *H. illucens*–microbial systems should be regarded as cornerstone technologies for a new circular bioeconomic industry. They address waste management, protein and fertilizer supply, and climate mitigation simultaneously. With coherent regulatory frameworks, targeted financial instruments, and sustained technological innovation, BSF–microbial platforms could transform organic waste from an environmental burden into a driver of inclusive and sustainable economic development.

## Generative-AI declaration

The Large Language Model ChatGPT 5.2 (OpenAI) was used to correct text written in English and occasionally to translate text from French into English.
